# A molecular platform for the diagnosis of multidrug-resistant and pre-extensively drug-resistant tuberculosis based on single nucleotide polymorphism mutations present in Colombian isolates of *Mycobacterium tuberculosis*

**DOI:** 10.1590/0074-02760150306

**Published:** 2016-02

**Authors:** Luz Maira Wintaco Martínez, Gloria Puerto Castro, Martha Inírida Guerrero

**Affiliations:** Instituto Nacional de Salud, Dirección de Investigación en Salud Pública, Grupo de Micobacterias, Bogotá, Colombia

**Keywords:** tuberculosis, multidrug-resistant tuberculosis, extensively drug-resistant tuberculosis, molecular diagnostic, mutations, SNP, reverse hybridisation

## Abstract

Developing a fast, inexpensive, and specific test that reflects the mutations present in *Mycobacterium tuberculosis* isolates according to geographic region is the main challenge for drug-resistant tuberculosis (TB) control. The objective of this study was to develop a molecular platform to make a rapid diagnosis of multidrug-resistant (MDR) and extensively drug-resistant TB based on single nucleotide polymorphism (SNP) mutations present in the*rpoB*, *katG*, *inhA*,*ahpC*, and *gyrA* genes from Colombian *M. tuberculosis* isolates. The amplification and sequencing of each target gene was performed. Capture oligonucleotides, which were tested before being used with isolates to assess the performance, were designed for wild type and mutated codons, and the platform was standardised based on the reverse hybridisation principle. This method was tested on DNA samples extracted from clinical isolates from 160 Colombian patients who were previously phenotypically and genotypically characterised as having susceptible or MDR *M. tuberculosis*. For our method, the kappa index of the sequencing results was 0,966, 0,825, 0,766, 0,740, and 0,625 for*rpoB*, *katG*, *inhA*,*ahpC*, and *gyrA,* respectively. Sensitivity and specificity were ranked between 90-100% compared with those of phenotypic drug susceptibility testing. Our assay helps to pave the way for implementation locally and for specifically adapted methods that can simultaneously detect drug resistance mutations to first and second-line drugs within a few hours.

During 2012, 8.6 million people became ill with tuberculosis (TB) worldwide, of which 450,000 developed multidrug-resistant TB (MDR-TB). Among untreated patients, MDR-TB constituted 3.6% and 20% of previously treated patients. At that time, 92 countries had reported at least one case of extensively resistant TB (XDR-TB) (WHO 2013).

The MDR-TB proportion in previously untreated patients in Colombia increased from 1.5% in 2000 to 2.4% in 2006 (Garzón et al. 2008), which provides ample reason to warn healthcare professionals and emphasises the need to develop an assay for the rapid diagnosis of drug-resistant TB. In addition, according to the Epidemiological Surveillance System of the National Institute of Health (INS) (INS 2013), in the last three years, 110 cases of MDR-TB per year on average have been identified, and 10 cases of XDR-TB have confirmed (INS 2013).

In 2006, the World Health Organization (WHO) formulated a policy for the management of MDR/XDR-TB, where the improvement of diagnosis is recommended (WHO 2006). Additionally, the Special Programme for Research and Training in Tropical Diseases presented needs for TB research in the world, and it recommended the development of diagnostic tests that are rapid, easy to perform, and produce accurate results for both types of drug-resistant TB (WHO/UNICEF/UNDP/World Bank/WHO Special Programme for Research and TDR 2013).

*Mycobacterium tuberculosis* resistant to the drugs used for its control is the result of the presence of mutations in specific regions of the genome (tbdreamdb.com/). Thus, sequencing is the gold standard method for finding mutations associated with drug resistance. However, sequencing cannot be used in all countries due to high cost. Therefore, the development of faster and cheaper in-house techniques to identify these mutations, for example, techniques based on hybridisation, has gained worldwide importance (García de Viedma 2003). Given this context, the development of rapid, inexpensive, and specific tests that reflect the mutations present in *M. tuberculosis* isolates according to geographic region is the main challenge for the control of drug-resistant TB.

Around the world, various methodologies have been developed to determine the phenotypic susceptibility to first and second-line drugs. The gold standard is the multiple proportions method, which is performed on solid medium and has the disadvantage of not producing results until four-six weeks after the primary isolation (Canetti et al. 1963).

Additionally, there are commercial, rapid phenotypic methods, such as BACTEC MGIT 960 (Becton Dickinson Diagnostic Systems), based on bacterial growth in liquid medium in the presence of the antibiotics that show good agreement with the multiple proportions method (Piffardi et al. 2004). Other rapid phenotypic methods include the colorimetric nitrate reductase assay, which shows good sensitivity (99%) and specificity (100%) for resistance to rifampicin (RMP) (Bwanga et al. 2009).

The MTT colorimetric test based on the oxidation-reduction of methyl thiazol tetrazolium presented a sensitivity of 89% and specificity of 96% (Martin et al. 2005). The microscopic observation drug susceptibility assay of the growth of *M. Tuberculosis* demonstrates 96% sensitivity and 96% specificity (Caviedes et al. 2000).

On the other hand, genotypic tests have been developed both in-house and commercially based on the identification of mutations associated with resistance, such as the INNO-LiPA-Rif.TB^®^ (Innogenetics NV, Belgium) and GenoType^®^ MTB DR plus (Hain Lifescience GmbH, Germany), which has a sensitivity and specificity above 90% for rifampin (RIF) (Viveiros et al. 2005,Ling et al. 2008, Bwanga et al. 2009, Skenders et al. 2011). The Xpert MTB/RIF (Cepheid) test, recommended by WHO in 2011 with 98.2% of sensitivity for smear-positive TB and 72.5% for smear-negative T, has a specificity of 99.2% when used on three specimens (Boehme et al. 2011).

Published studies from various parts of the world have used methodology based on probes and reverse hybridisation to identify RMP resistance: Morcillo et al. (2002) found a sensitivity of 92.8% for mutations in*rpoB*, Mokrousov et al. (2004) conducted a multicentre study to detect resistance to RMP, isoniazid (INH), streptomycin (SM), and ethambutol (EMB), with good results, Senna et al. (2006) developed an assay for detecting mutations in*rpoB* and found a sensitivity of 93% and specificity of 100%, andSuresh et al. (2006) reports a concordance of 98% with respect to sequencing. In Colombia, Hernandez-Neuta et al. (2010) published an assay for mutations defining MDR-TB in the *rpoB*, *katG*, and *inhA*genes with sensitivity and specificity greater than 90% for both INH and RIF.

Moreover, trials have been developed based on macroarrays as described by Zhang et al. (2007), who used probes to determine mutations in *rpoB*, *katG*, *inhA*, and*ahpC* to define MDR-TB.

For *M. tuberculosis* susceptibility to second-line drugs, Antonova et al. (2008) developed reverse hybridisation to fluoroquinolones (FQ) based on *gyrA* gene mutations with a sensitivity of 94%, and Giannoni et al. (2005) developed methods with 100% sensitivity.

The purpose of this study was to develop a molecular tool for rapid detection of MDR TB and extensively drug-resistant TB based on the mutations in the*rpoB*, *katG*, *inhA*,*ahpC*, and *gyrA* genes by reverse hybridisation of membranes. The results of the sequencing and phenotypic simplified proportion method and BACTEC MGIT 960 were used as the reference methods and were performed on the same set of 160 samples of *M. tuberculosis* isolated from Colombian patients during the last 10 years.

## SUBJECTS, MATERIALS AND METHODS

*Clinical isolates* - For this study, 160 *M. tuberculosis* isolates belonging to the biobank from the Mycobacteria Laboratory of the INS in Bogotá, Colombia, were used. These were isolated from Colombian patients during 2000-2010. Of these isolates, 130 were MDR and 30 were pansusceptible.

Reference strains *M. tuberculosis* ATCC 25618 H37RV (pansusceptible),*M. tuberculosis* ATCC 35822 (resistant to INH), ATCC 35838 (resistant to RMP), and Colombian clinical isolates with an XDR phenotype were used as controls for the microbiological and genetics tests.

The stored isolates were subcultured on Lowenstein-Jensen solid medium and incubated at 37ºC for two-four weeks. These samples were used only for tests of analytical sensitivity of hybridisation and sequencing developed here.

*Ethics statement* - All study procedures were approved by the INS Ethical Committee in Research. According to the 8430-1993 resolution from the Colombian Ministry of Health, informed consent was not required because all samples used in this study were the isolates obtained from the biobank of the mycobacteria group without any personal information about patients, i.e., they were anonymous samples.

*Drug susceptibility testing (DST)* - Previous to introduction of these isolates to the biobank, phenotypic susceptibility to the first-line DST for RMP, INH, SM, and EMB was performed using the simplified proportion method (Canetti et al. 1963) and the resistance to ofloxacin, levofloxacin, and moxifloxacin were determined using the BACTEC MGIT 960 automated system, according to the manufacturer’s instructions.

*DNA extraction* - The strains were subcultured on Lowenstein-Jensen solid medium during four weeks at 37ºC and then suspended in 400 µL of tris-borate-ethylenediamine tetraacetic acid (EDTA) 0.1X in an Eppendorf tube. The cells were heat-killed at 99ºC for 30 min. The DNA extraction was performed according to the recommendations of van Soolingen et al. (2002).

*PCR amplification* - The amplification of the fragments of interest for each molecular target was standardised in a final volume of 50 µL containing 0,1-1,2 μL of 25 μM biotin-labelled forward primers for*rpoB*, *katG*, *inhA*,*ahpC*, *and gyrA*; the same quantity of 25 μM reverse primers (Table I) and 1 μL DNA sample were also included.

**TABLE I:** Sequence of the primers for the amplification of target gene fragments in *rpoB*, *katG*,*inhA*, *ahpC*, and*gyrA*

Drug	*Locus*	Primers	Primer sequence	Amplicon length (bp)
Rifampicin	*rpoB*	*rpoB-F*	5’GACGACCATCGACCACTTC3’	616
		*rpoB-R*	5’AGGGCACGTACTCCACCTC3’	
Isoniazid	*katG*	*katG-F*	5’GGGACATCGAGGAAGTGATG3	1,516
		*katG-R*	5’GATTCCACGTCGGTTTGTTC3’	
	*inhA*	*inhA-F*	5’GGCAAACGGATTCTGGTTA3’	765
		*inhA-R*	5’GTCGGCGTAGATGATGTCAC3’	
	*ahpC*	*ahpC-F*	5’GCTAACCATTGGCGATCAA3’	490
		*ahpC-R*	5’GTCGAGCACTCGCAGTACCT3’	
Fluoroquinolones	*gyrA*	*gyrA-F*	5’CGCAGCTACATCGACTATGC3’	322
		*gyrA-R*	5’GGGCTTCGGTGTACCTCAT3’	

The cycling conditions were as follows: denaturation at 95ºC for 5 min, 40 cycles of denaturation at 95ºC for 30 s, annealing at 64ºC for 30 s, and extension at 72ºC for 30 s, then 40 cycles and final extension step at 72ºC for 5 min. Each amplification experiment included a positive (*M. tuberculosis H37Rv* DNA, 100 ng) and a negative control (water). For standardisation of the amplification reactions, the polymerase chain reaction (PCR) products were analysed using agarose gel electrophoresis followed by detection in ultraviolet light after staining with ethidium bromide and comparison of the amplicons to a molecular weight marker.

*Development of molecular tool capture oligonucleotide design* - The capture oligonucleotides were designed with the wild-type (WT) and mutated sequences according to the mutations found in the Colombian isolates in the*rpoB*, *katG*, *ahpC*,*inhA*, and *gyrA* genes. The melting temperatures were calculated and the secondary structures of the designed oligonucleotides were estimated using an OligoAnalyzer (Integrated DNA Technologies (eu.idtdna.com/analyzer/Applications/OligoAnalyzer/). The lengths of the oligonucleotides were adjusted to maintain the difference in the melting temperatures within 2-3ºC and were modified with amine groups at the 5’end, which were synthesised by Invitrogen (Table II).

**TABLE II:** Sequence of the capture oligonucleotides sequence used for development of the molecular platform according to the studied codon

Name	Capture oligonucleotide sequence	Codon studied
*rpoBWT511*	5’AMINO-GGC-ACC-AGC-CAG-CTG-AGC-3’	511
*rpoBMUT511*	5’AMINO-GGC-ACC-AGC-CAG-CCG-AGC-3’	511
*rpoBWT513*	5’AMINO-ACC-AGC-CAG-CTG-AGC-CAA-TTC-3’	513
*rpoBMUT513GAA*	5’AMINO-ACC-AGC-CAG-CTG-AGC-GAA-TTC-3’	513
*rpoBMUT513CCA*	5’AMINO-ACC-AGC-CAG-CTG-AGC-CCA-TTC-3’	513
*rpoBWT516*	5’AMINO-TTC-ATG-GAC-CAG-AAC-AAC-CCG-3’	516
*rpoBMUT516TAC*	5’AMINO-TTC-ATG-TAC-CAG-AAC-AAC-CCG-3’	516
*rpoBMUT516GTC*	5’AMINO-TTC-ATG-GTC-CAG-AAC-AAC-CCG-3’	516
*rpoBMUT516GAG*	5’AMINO-TTC-ATG-GAG-CAG-AAC-AAC-CCG-3’	516
*rpoBWT522*	5’AMINO-CCG-CTG-TCG-GGG-TTG-ACC-3’	522
*rpoBMUT522*	5’AMINO-CCG-CTG-TTG-GGG-TTG-ACC-3’	522
*rpoBWT526*	5’AMINO-TTG-ACC-CAC-AAG-CGC-CGA-3’	526
*rpoBMUT526GAC*	5’AMINO-TTG-ACC-GAC-AAG-CGC-CGA-3’	526
*rpoBMUT526TAC*	5’AMINO-TTG-ACC-TAC-AAG-CGC-CGA-3’	526
*rpoBMUT526AAC*	5’AMINO-TTG-ACC-AAC-AAG-CGC-CGA-3’	526
*rpoBMUT526CTC*	5’AMINO-TTG-ACC-CTC-AAG-CGC-CGA-3’	526
*rpoBMUT526CAG*	5’AMINO-TTG-ACC-CAG-AAG-CGC-CGA-3’	526
*rpoBWT531*	5’AMINO-CTG-TCG-GCG-CTG-GGG-CCC-GGC-3’	531
*rpoBMUT531TTG*	5’AMINO-CTG-TTG-GCG-CTG-GGG-CCC-GGC-3’	531
*rpoBMUT531TGG*	5’AMINO-CTG-TGG-GCG-CTG-GGG-CCC-GGC-3’	531
*katGWT138*	5’AMINO-TGG-CCC-GAC-AAC-GCC-AGC-TTG-3’	138
*katGMUT138CAC*	5’AMINO-TGG-CCC-GAC-CAC-GCC-AGC-TTG-3’	138
*katGMUT138GAC*	5’AMINO-TGG-CCC-GAC-GAC-GCC-AGC-TTG-3’	138
*katGMUT138AGC*	5’AMINO-TGG-CCC-GAC-AGC-GCC-AGC-TTG-3’	138
*katGWT315*	5’AMINO-GGT-AAG-GAC-GCG-ATC-ACC-AGC-3’	315
*katGMUT315ACC*	5’AMINO-GGT-AAG-GAC-GCG-ATC-ACC-ACC-3’	315
*katGMUT315AAC*	5’AMINO-GGT-AAG-GAC-GCG-ATC-ACC-AAC-3’	315
*katGMUT315ATC*	5’AMINO-GGT-AAG-GAC-GCG-ATC-ACC-ATC-3’	315
*katGMUT315AGG*	5’AMINO-GGT-AAG-GAC-GCG-ATC-ACC-AGG-3’	315
*katGMUT315ACA*	5’AMINO-GGT-AAG-GAC-GCG-ATC-ACC-ACA-3’	315
*katGWT316*	5’AMINO-GGC-ATC-GAG-GTC-GTA-TGG-3’	316
*katGMUT316GAC*	5’AMINO-GAC-ATC-GAG-GTC-GTA-TGG-3’	316
*inhAWT21*	5’AMINO-TCG-TCG-ATC-GCG-TTT-CAC-ATC-3’	21
*inhAMUT21GTC*	5’AMINO-TCG-TCG-GTC-GCG-TTT-CAC-ATC-3’	21
*inhAMUT21ACC*	5’AMINO-TCG-TCG-ACC-GCG-TTT-CAC-ATC-3’	21
*inhAWT94*	5’AMINO-CTC-GAC-GGG-GTG-GTG-CAT-TCG-3’	94
*inhAMUT94GCG*	5’AMINO-CTC-GAC-GGG-GTG-GTG-CAT-GCG-3’	94
*inhAMUT94ACG*	5’AMINO-CTC-GAC-GGG-GTG-GTG-CAT-ACG-3’	94
*ahpCWT44*	5’AMINO-GAC-GAA-CAC-CCA-GGC-AAG-TGG-3’	44
*ahpCMUT44CGA*	5’AMINO-GAC-GAA-CAC-CGA-GGC-AAG-TGG-3’	44
*ahpCWT48*	5’AMINO-CGG-GTG-GTG-TTC-TTT-TGG-CCG-3’	48
*ahpCMUT48CGC*	5’AMINO-CGC-GTG-GTG-TTC-TTT-TGG-CCG-3’	48
*gyrAWT61*	5’AMINO-GAT-TCC-GGC-TTC-CGC-CCG-GAC-3’	61
*gyrAMUT61AAT*	5’AMINO-AAT-TCC-GGC-TTC-CGC-CCG-GAC-3’	61
*gyrAMUT61GAG*	5’AMINO-GAG-TCC-GGC-TTC-CGC-CCG-GAC-3’	61
*gyrAMUT61GAA*	5’AMINO-GAA-TCC-GGC-TTC-CGC-CCG-GAC-3’	61
*gyrAMUT61GCT*	5’AMINO-GCT-TCC-GGC-TTC-CGC-CCG-GAC-3’	61
*gyrAMUT61GGT*	5’AMINO-GGT-TCC-GGC-TTC-CGC-CCG-GAC-3’	61
*gyrAWT74*	5’AMINO-CGC-AGC-CAC-GCC-AAG-TCG-GCC-3’	74
*gyrAMUT74GCA*	5’AMINO-CGC-AGC-CAC-GCC-AAG-TCG-GCA-3’	74
*gyrAMUT74GCG*	5’AMINO-CGC-AGC-CAC-GCC-AAG-TCG-GCG-3’	74
*gyrAWT94*	5’AMINO-GGC-GAC-GCG-TCG-ATC-TAC-GAC-3’	94
*gyrAMUT94AAC*	5’AMINO-GGC-GAC-GCG-TCG-ATC-TAC-AAC-3’	94
*gyrAMUT94GGC*	5’AMINO-GGC-GAC-GCG-TCG-ATC-TAC-GGC-3’	94
*gyrAMUT94GCC*	5’AMINO-GGC-GAC-GCG-TCG-ATC-TAC-GCC-3’	94
*gyrAMUT94TAC*	5’AMINO-GGC-GAC-GCG-TCG-ATC-TAC-TAC-3’	94
*gyrAMUT94CAC*	5’AMINO-GGC-GAC-GCG-TCG-ATC-TAC-CAC-3’	94
*gyrAWT100-102*	5’AMINO-GCC-CAG-CCC-TGG-TCG-CTG-CGC-3’	100.102

*Membrane preparation* - First, 20 x 20 cm Biodyne-C^®^membranes (Pall Corp) were cut and activated with 15 mL of 16% EDAC at room temperature (RT). After washing twice with deionised water, the membranes were placed over an absorbent carrier and mounted in Miniblotter-45 (Immunetics). The WT and mutated capture oligonucleotides were diluted in 150 µL of 0.5 M NaHCO_3_ pH 8.4 and placed in channels of the mini blotter. They were then reacted with the membrane to allow irreversible binding at RT. Subsequently, the excess solution was removed by suction, and the membrane was washed with 200 mL of 0.1 M NaOH followed by washing with deionised water for 2 min. Two final washes were performed: one in 250 mL of 2x SSPE + 0.1% SDS at 50ºC and one with 100 mL of 20 mM EDTA, pH 8.0 at RT. Each membrane was marked at the ends with India ink diluted 1:100 in 2X SSPE. The membranes were stored in 20 mM EDTA, pH 8.0 at 40ºC until use (de Beenhouwer et al. 1995).

*Optimisation of membrane conditions* - For each molecular target,*rpoB*, *katG*, *ahpC*,*inhA*, and *gyrA,* membranes were prepared in concentrations of 12.5 pmol, 50 pmol, 100 pmol, and 200 pmol of each of the capture oligonucleotides. Then, 10 µL, 15 µL, and 20 µL of DNA products from the control strains, amplified by PCR and labelled with biotinylated forward 5’primer, were also tested. Hybridisation temperatures of 50ºC, 52ºC, and 54ºC were tested. The development was optimised in the X-ray film exposed in a hyper cassette for 1, 15, 30, 60, and 120 min.

*Reverse hybridisation on the definitive membranes and protocol* - Using the optimal conditions of concentration of capture oligonucleotides, volume of the PCR product, annealing temperature, and exposure time for each of the studied molecular targets (Table III), the evaluation of the 160 samples was performed. The results were recorded in Excel tables designed for this purpose, after scanning the autoradiographic signal in the films obtained. Briefly, labelled PCR products were diluted in 150 µL of buffer 2x SSPE + 0.1% SDS and denatured to 100ºC for 10 min, cooling on ice immediately. Denatured products were applied to each membrane placed in a mini blotter, with the first and last channel filled with 2x SSPE buffer. Each membrane was allowed to hybridise for 1 h at the optimum temperature and was washed twice with 2x SSPE + 0.5% SDS at 55ºC for 20 min. Then, 2.5 µL of streptavidin-peroxidase conjugate (500 U/mL) was added (Roche^®^) in 10 mL of 2x SSPE + 0.5% SDS buffer preheated to 42ºC and incubated by rotation for 45 min. The membranes were washed twice with 2x SSPE buffer at RT. Detection was performed by mixing 10 mL of reagent 1 and 2 of ECL chemiluminescence kit detection (GE Healthcare) and exposing the X-ray film on a hyper cassette during the optimum time for each target (de Beenhouwer et al. 1995).

**TABLE III:** Optimal conditions of reverse hybridisation for each molecular target studied

Standardised parameter	Oligonucleotides target				
	*rpoB*	*katG*	*ahpC*	*inhA*	*gyrA*
PCR product used (µL)	15	20	15	15	25
Oligonucleotides [ ]^*a*^ pmol used	50-100	50-100	50-100	50-100	50-100
Hibridisation temperature (ºC)	52	54	54	54	54
Hibridistion time (h)	1	1	1	1	1
RX exposition time	1 h-ON	1 h-ON	1 h-ON	1 h-ON	1 h-ON

*a*: concentration in pmol; PCR: polymerase chain reaction.

*Reproducibility of detection of mutations by reverse hybridisation* - To obtain our analysis results, we conducted triplicate assays at three different times.

*Sequencing* - The fragments of the genes that determine resistance (i.e., *rpoB*, *katG*, *inhA*,*ahpC*, and *gyrA*) were amplified using the corresponding primers (Table I) to 160 samples and then subjected to capillary-sequencing using one of the terminal primers and the ABI PRISM^®^ BigDye™ Terminator v.3.1 (Applied Biosystems, USA) followed by analysis with the 3730 automatic DNA analyzer.

*Statistical analysis* - Statistical analysis was performed with the OpenEpi v.3.0 package. Parameter assessment was performed using a diagnostic test as a gold standard for each of the targets studied for each drug, the phenotypic susceptibility results, and sequencing.

## RESULTS

*Membranes and protocol to definitive reverse hybridisation* - Figure shows an autoradiograph of the distribution and the reverse hybridisation results from developed membranes.

*Detection of mutations by reverse hybridisation* - The procedure consisted of the following steps: (i) PCR *rpoB*,*katG*, *inhA*, *ahpC*, and*gyrA gene* fragments*,* (ii) hybridisation of biotin-labelled single-stranded PCR products on the membrane, and (iii) detection by autoradiography.

The hybridisation pattern corresponding to *M. tuberculosis* H37Rv WT DNA is shown in Figure.

**Figure:**
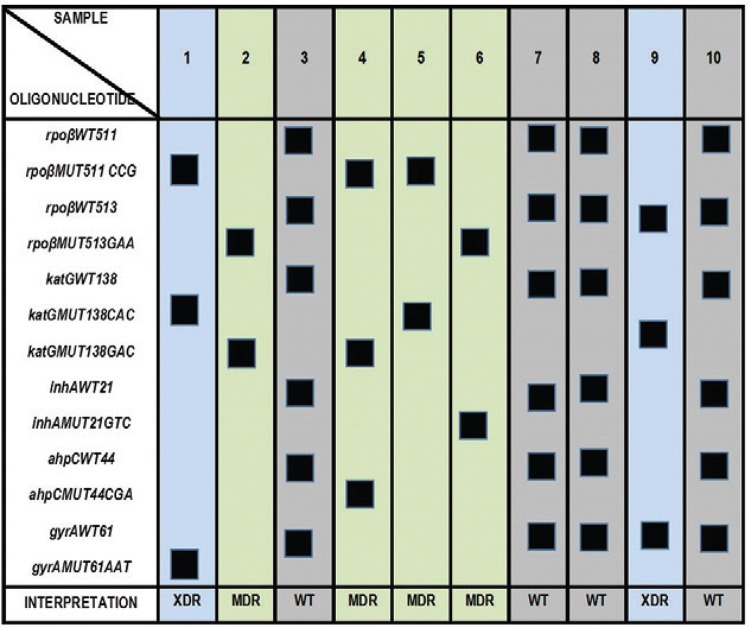
Schematic view of the distribution and the reverse hybridisation results from developed membranes. MDR: multidrug-resistant; WT: wild-type; XDR: extensively resistant.

*Reproducibility of mutation detection by reverse hybridisation* - The same results were obtained each of the three times the tests were performed. The interassay and intralaboratory reproducibility, with a single operator and a single reader, was 100%.

*Correlation of phenotypic DST with molecular assays developed* - Data obtained from the reverse hybridisation membrane analysis and DST data for 160 strains and each molecular target are shown (Table IV).

**TABLE IV:** Operative characteristics of reverse hybridisation membrane using phenotypic drug susceptibility testing as gold standard test

Drugs	*Locus*	Operative characteristics					
		Sensibility	Specificity	PPV	PNV	Diagnostic precision	Kappa index
Rifampicin	*rpoB*	0.80	1.0	1.0	0.63	0.85	0.669
Isoniazid	*katG*	0.72	1.0	1.0	0.64	0.81	0.631
	*ahpC*	0.09	0.97	0.92	0.20	0.26	0.023
	*inhA*	0.07	0.97	0.91	0.20	0.25	0.017
Fluoroquinolones	*gyrA*	0.11	0.96	0.92	0.16	0.23	0.017

PNV: predictive negative value; PPV: predictive positive value.

*rpoB* mutations conferring RMP resistance were found in 80% (104 of 130 strains) of the samples with resistance phenotype (MDR). No mutations in*rpoB* conferring RMP resistance were found in any of the samples with a sensitive phenotype (44 strains).

*katG* mutations conferring INH resistance were found in 72.2% (44 of 61 strains) of the samples with a resistance phenotype (MDR). No mutations in*katG* conferring INH resistance were found any of the samples with a sensitive phenotype (30 strains).

*inhA* mutations conferring INH resistance were found in 7.7% (10 of 130 strains) of the samples with resistance phenotype (MDR). No mutations in*inhA* conferring INH resistance were found in 96.7% (29 of 30 strains) of the samples with a sensitive phenotype.

*ahpC* mutations conferring INH resistance were found in 9.2% (12 of 130 strains) of the samples with a resistance phenotype (MDR). No mutations in*ahpC* conferring INH resistance were found in 96.7% (29 of 30 strains) of the samples with a sensitive phenotype.

*gyrA* mutations conferring FQ resistance were found in 10.2% (12 of 118 strains) of the samples with a resistance phenotype (MDR). No mutations in*gyrA* conferring FQ resistance were found in 95.3% (20 of 21 strains) of the samples with a sensitive phenotype.

*Correlation of sequencing results with molecular assays developed* - Data obtained from the reverse hybridisation membrane analysis and sequencing data for 160 strains and each molecular target are shown (Table V).

**TABLE V:** Operative characteristics of reverse hybridisation membrane using sequencing as gold standard test

Drugs	*Locus*	Operative characteristics					
		Sensibility	Specificity	PPV	PNV	Diagnostic precision	Kappa index
Rifampicin	*rpoB*	0.977	1.0	1.0	0.957	0.985	0.966
Isoniazid	*katG*	0.875	0.954	0.955	0.872	0.912	0.825
	*ahpC*	0.900	0.973	0.692	0.993	0.968	0.766
	*inhA*	1.00	0.955	0.615	1.0	0.958	0.740
Fluoroquinolones	*gyrA*	0.599	0.975	0.769	0.944	0.927	0.625

PNV: predictive negative value; PPV: predictive positive value.

*rpoB* mutations conferring RMP resistance were found in 97.7% (127 of 130 strains) of the samples with mutation in sequencing. No mutations in*rpoB* conferring RMP resistance were found in any of the samples without mutation in sequencing (67 strains).

*katG* mutations conferring INH resistance were found in 87.5% (42 of 48 strains) of the samples with mutation in sequencing. No mutations in*katG* conferring INH resistance were found in 95.4% (41 of 43 strains) of the samples without mutation in sequencing.

*inhA* mutations conferring INH resistance were found in 100% (8 of 8 strains) of the samples with mutation in sequencing. No mutations in *inhA*conferring INH resistance were found in 96.7% (107 of 112 strains) of the samples without mutation in sequencing.

*ahpC* mutations conferring INH resistance were found in 90% (9 of 10 strains) of the samples with mutation in sequencing. No mutations in *ahpC*conferring INH resistance were found in 97.4% (146 of 150 strains) of the samples without mutation in sequencing.

*gyrA* mutations conferring FQ resistance were found in 59% (10 of 17 strains) of the samples with mutation in sequencing. No mutations in *gyrA*conferring FQ resistance were found in 97.5% (117 of 120 strains) of the samples without mutation in sequencing.

The kappa index, or degree of agreement between the developed test and sequencing, qualifies as good and very good classification according to Landis and Koch (1977) because it ranged from 0.875-1.0 (Table V).

## DISCUSSION

The development of a rapid, inexpensive, and specific test that reflects mutations present in *M. tuberculosis* isolates, according to geographic region, is the main challenge for the control of drug-resistant TB. We designated the present test based on two previous published methods on which it was based, rifoligotyping (de Beenhouwer et al. 1995,Kremer et al. 1997), and we developed a reverse hybridisation in-house technique that assesses four molecular targets for single nucleotide polymorphism (SNP) detection as resistance predictors of TB-MDR. This test allowed us to seamlessly and simultaneously perform*rpoB*, *katG*, *inhA*,*ahpC*, and *gyrA* mutation detection for MDR-TB and pre XDR-TB by genotypic detection testing.

Compared with sequencing (molecular platform versus sequencing), both the sensitivity and specificity were ranked 1.0-0.599 for the prediction of resistant strains (MDR/XDR). The sensitivity decreased to 0.07 when phenotypic DST was used as the criterion because mutations in *ahpC*, *inhA*, or*gyrA* of MDR clinical isolates from the Colombian collection were scarce.

Sequencing showed that our MDR strain had a SNP in the *rpoB* RMP resistance-determining region, which was in contrast with the commercial methods. This was corroborated by our test results because we included codons 511, 513, and 522 in the study, as well as other mutations in 516,526 and 531, which are more frequent in Colombian isolates, but not included in the commercial tests that are currently used.

Furthermore, the sequencing showed that our MDR strains had SNPs in the*katG* codons (other than 315) at a considerable proportion, necessary to correctly identify the Colombian MDR strains. These SNPs were also included in our platform; however, only half of the MDR isolates had mutations in this gene, as found in studies from other countries (TBDR 2011).

At this point, the frequencies of different mutations in drug resistance genes with respect to geographic origins of *M. tuberculosis* isolates have been described exhaustively in various studies, and a specific database was created (Sandgren et al. 2009). With this study, we demonstrated that the local predominance of a specific mutation is possible, but larger worldwide studies are necessary to better understand the geographical distribution of resistance mutations.

The predictive positive value of the developed test ranged from 0.615-1.0, while the predictive negative value was between 0.872-1.0 when compared with sequencing. It is well known that the predictive values of a test directly depend on the prevalence of the characteristic being studied in a particular population.

Therefore, in a given population with features of TB similar to Colombia, it might be economically beneficial to introduce our test. Such introduction would significantly improve the cost-effectiveness of MDR-TB control. This test could become a diagnostic strategy in countries with high prevalence rates of MDR-TB, as a complement or alternative to the other commercial assays.

Compared with commercial tests, our line-probe assay is very reliable in terms of operationalisation because this format allows the execution of 40 samples at once. In 8 h of work, a worker can perform two procedures (80 samples). Therefore, the cost (≈39 USD/test) provides potential advantages for application in centralised laboratories or in smaller laboratories. We believe that the test should be performed in biosafety level 3 laboratories, where biosecurity and staff capability ensure that the test performance would be equal to what we have achieved. However, it could be performed in biosafety level 2 laboratories with a biological safety cabinet and capable staff.

The applicability of mutation detection using our molecular platform of reverse hybridisation is clearly useful in the services network context, where the cost/benefit of the most complex procedures is restricted to the most developed network levels.

As with many other researchers in the field of XDR, we did not find clear markers in our sequencing of *tlyA* and *rrs*, and therefore, we restrict ourselves to *gyrA*, which is considered a marker of pre-XDR strains. This is one limitation of our method.

In conclusion, we have developed a molecular test with good sensitivity and specificity that is useful for identifying MDR-TB and pre-XDR TB in the Colombian population. This could have a significant impact on the early diagnosis of resistance and may contribute to strategies for controlling resistant TB in the country.
